# Diagnosis and surgical management of congenital intestinal malrotation presenting with midgut volvulus in an adult: high index of suspicion (case report)

**DOI:** 10.11604/pamj.2018.29.154.13910

**Published:** 2018-03-15

**Authors:** Gezahen Negusse Ayane, Khutsafalo Kadimo

**Affiliations:** 1 University of Botswana, Department of General Surgery and Referral Hospital Princes Marina, Gaborone, Botswana; 2 University of Botswana, Department of Library Services, Gaborone, Botswana

**Keywords:** Congenital intestinal malrotation, midgut volvulus, laparotomy, laparoscopic Ladd´s, gangrenous, anastomosis, case report

## Abstract

Congenital intestinal malrotation is a gastrointestinal anomaly whose most serious complication is midgut volvulus. More commonly, it presents as an incidental finding at laparotomy, or as a finding on diagnostic imaging (Ultrasound, CT, Upper GI contrast study). Most patients are diagnosed in childhood. Laparoscopic Ladd's procedure is an accepted alternative to Laparotomy in children but has not been well-studied in adult. We present the case of this unexpected finding in a patient 38 years old, during emergency laparotomy for mechanical intestinal obstruction. Intra-operative findings included intestinal malrotation with small bowel volvulus. The terminal ilea and cecum were gangrenous on the basis of ischemic necrosis. A limited right hemycolectomy and primary end-to- end anastomosis was performed.

## Introduction

Congenital Intestinal malrotation is a complex disorder caused by incomplete or abnormal rotation of the intestine during fetal development. Embryological development and anatomical variations were described in 1923 by Dott [1]. The intestines start to grow in the fourth week of gestation. Physiological increase in intestinal length occurs and the bowel herniates into the umbilical cord. The hernia is reduced in the 10^th^ week of gestation, with the bowel rotating in an anticlockwise direction, and the caecum settles in its typical right lower quadrant position at 12^th^ week. Intestinal malrotation is a disorder resulting from the lack of fetal intestinal physiological rotation [1]. There are often fibrous Ladd's bands that tether the cecum and right colon to the right abdominal wall. These may also contribute to the obstructive symptoms, over and above the twisting of the mesentery. Intestinal malrotations comprise various anatomic anomalies ranging from complete nonrotation to normal position and broad-based mesentery. Intestinal rotational anomalies are classified according to the anatomical variation, and include incomplete rotation, mixed rotation, atypical malrotation, and variants of malrotation. They can be categorized into two groups: typical and atypical malrotation based on the position of the ligament of Treitz according to the right and left of the midline, respectively. Intestinal malrotation occurs in approximately 0.2% of all births. Symptoms usually occur in the early weeks of life, and the malrotations are generally diagnosed during this period. More than 40% of intestinal malrotations are diagnosed within 1 week after birth and 75-85 % within a year after birth. Although the precise incidence of intestinal malrotation is unknown, it is estimated that it occurs between 0.0001 % and 0.19 % of adults [1-3]. Generally, intestinal malrotation is incidentally found in adults due to its asymptomatic or nonspecific presentation with mild symptoms. In the present paper we present a case of an adult 38 years old female patient with intestinal malrotation with volvulus leading to ischemic necrosis and gangrene of the ileo-caecal area. Limited right hemi colectomy and primary end-to- end anastomosis was done. The patient was discharged from the ward 6 days post operatively. Follow up fifteen days and three months post discharge revealed full recovery.

## Patient and observation

This is a case report of 38 year old female patient, HIV negative, with long standing intermittent chronic abdominal pain. She had no others chronic medical illness. Previous surgical history consisted of Cesarean section 10 years ago. She presented to the Accident and Emergency department of Princess Marina Hospital (PMH), complaining of colicky abdominal pain and abdominal distension for previous 5 days, as well bilious vomiting and inability to pass stool or flatus for the same 5 days. Vitals signs were BP: 127/76 Torr; pulse 125/ min; respiratory rate: 20/ min; and temperature: 36.5 c. Basic blood investigation full blood counts, and electrolytes) are normal. Chest -X- Ray: normal and Abdominal X- Ray (AP- Erect); show multiple central air-fluid levels. The patient then was taken for emergency exploratory laparotomy with working diagnosis of intestinal obstruction secondary to intra-abdominal adhesion; the finding was intestinal malrotation and midgut volvulus. The patient had gangrene of the terminal ileus and caecum. No Ladd's bands were found. No intra-abdominal adhesions or adhesive bands were found. Limited right hemi-colectomy and primary end-to-end anastomosis was done. Her post-operative course was uneventful, she was discharged from the ward after 6 days. Follow up at fifteen days and three months revealed that the patient was doing well.

## Discussion

The intestines are classified into three groups based on the origin of the arterial supplies: foregut, midgut, and hindgut. The duodenum, ileum, jejunum, caecum, and ascending colon constituting two-third of the proximal part of the transverse colon are supplied from the superior mesenteric artery (SMA). Intestinal rotation is completed within 4- 12 weeks of intrauterine life. The rapid increase in length of the intestine and physiologic herniation into the umbilical cord occurs in the fifth week, a 270^o^ anticlockwise rotation along the SMA axis and the return of hernia back into the abdominal cavity occur in to 10th week, and the location of the caecum in the right lower quadrant are completed in the 12^th^ week. The variation between the normal rotation and failure of the intestine to rotate due to any malfunction in this process are known as malrotation [1,4]. There are several types of rotational anomalies: a diversity of anatomic configurations, ranging from a not-quite normal intestinal position to complete nonrotation [1]. The most common variations are nonrotation, reverse fixation, and malrotation. Stinger classified several types of malrotation according to the embryologic state of development. Type Ia is defined as nonrotation of the colon and duodenum, and Type IIa is defined as nonrotation of the duodenum only. Type IIb, the duodenum and colon show reversed rotation; Type IIc, there is only reversed rotation of the duodenum. Type IIIa, both the duodenum and colon are not rotated. Type IIIb is characterized by incomplete attachment of the hepatic flexure. Type IIIc, incomplete attachment of the caecum and Type IIId, is characterized by an internal hernia near the Ligament of Treitz [5]. The frequency of each anomaly is not known because some are asymptomatic and are found incidentally on imaging studies, surgery, or even autopsy. Malrotation of the midgut has usually been estimated to occur in approximately one in 500 newborns, and presents within the first month of life in 64-80% of patients. However, some patients will present later, even in adulthood, or remain asymptomatic for life. Intestinal malrotation in adult has an approximate incidence of 0.19 - 0.2% [3].

The symptoms in newborn infants are those of intestinal obstruction, such as bilious vomiting. Unlike the pediatric population, most adult patients with malrotation lack other congenital anomalies and free of symptoms related to their rotational anomaly. The minority of adults who do have symptoms typically present a prolonged history of abdominal complaints suggestive of obstruction. The most common symptoms found in adults is chronic abdominal pain, which leads clinicians and patients to attribute symptoms to the wrong diagnosis. All too often, such patients undergo numerous investigations and carry diagnostic labels such as irritable bowel syndrome, peptic ulcer disease, or psychogenic bowel disorder [6]. There are different modalities of investigations; upper gastrointestinal contrast with small bowel follow through (UGI/SBFT), barium enema, plain abdominal radiography, abdominal ultrasound and abdominal computed tomography (CT). Abdominal CT can show clearly intestinal position and, most importantly, the position of SMA in relationship to the SMV [1,6,7]. Surgery for incidentally detected asymptomatic cases is controversial because of the risk of volvulus and obstruction. However, surgery is recommended for patients with intestinal obstruction. Many case reports and original articles on intestinal malrotation might have missed duodenal obstruction on the basis of Ladd's bands [8]. The majority of adults with congenital intestinal malrotation and volvulus present with an acute abdomen, and even intestinal strangulation and necrosis [9]. Ladd's procedure with broadening of the mesentery, lysis of Ladd's bands, positioning the bowel in a position of non-rotation (all the small bowel on the right and all the large bowel on the left), and appendectomy can be successfully applied by either open laparotomy or laparoscopy. As mentioned above, the procedure involves four steps; (a)-counterclockwise detorsion or reduction of the volvulus, if present, (b) - division of the abnormal peritoneal bands (Ladd's bands) overlying the duodenum thereby relieving the cause of the intermittent obstruction,(c)-widening of the narrowed root of the small bowel mesentery by mobilizing the duodenum; and division of the adhesions around the SMA to prevent further volvulus, and placement of the small bowel to the right of the abdomen and the caecum to the left, and appendectomy[1,3]. Some authors state that the use of Ladd's procedure or division of Ladd's bands and adhesiolysis relieves symptoms and due to the formation of intra-abdominal adhesions after the procedure, prevents recurrence in the majority of patients. Conventional wisdom dictates that patients who have undergone laparotomy are at higher risk for forming post-operative adhesion than patients undergoing laparoscopy [10]. However, in the case of our patient, there were no Ladd's bands. We found midgut volvulus with gangrenous small bowel, and the resection of the gangrenous bowel and primary anastomosis was necessary.

## Conclusion

Adult congenital midgut volvulus often manifests with subtle obstructive symptomatology which can easily be misdiagnosed. A high index of suspicion is required. Doppler ultrasound and CT are helpful for preoperative diagnosis and positional crossing and whirlpool volvulus of SMA are the characteristic manifestation of congenital midgut malrotation[9]. Some controversy exists over the surgical management of rotational anomalies in asymptomatic or minimally symptomatic patients. Because the entire midgut is at risk, most surgeons would err on the side of surgical exploration, assuring that the small bowel mesentery is as broad as possible, and then completing the Ladd's procedure as described above. In the case of acute presentation, the entity must be quickly recognized, the bowel must be untwisted (usually in a counter-clockwise direction), and intestinal resection of necrotic bowel carried out if necessary. The remainder of the formal Ladd's procedure should then be completed.

## Competing interests

The authors declare no competing interests.
